# Individual differences in hyper-realistic mask detection

**DOI:** 10.1186/s41235-018-0118-3

**Published:** 2018-06-27

**Authors:** Jet G. Sanders, Rob Jenkins

**Affiliations:** Department of Psychology, University of York, Heslington, York, YO10 5DD UK

**Keywords:** Masks, Disguise, Face perception, Face detection, Face recognition, Deception, Fraud, Passports, Performance enhancement, Individual differences

## Abstract

Hyper-realistic masks present a new challenge to security and crime prevention. We have recently shown that people’s ability to differentiate these masks from real faces is extremely limited. Here we consider individual differences as a means to improve mask detection. Participants categorized single images as masks or real faces in a computer-based task. Experiment 1 revealed poor accuracy (40%) and large individual differences (5–100%) for high-realism masks among low-realism masks and real faces. Individual differences in mask categorization accuracy remained large when the Low-realism condition was eliminated (Experiment 2). Accuracy for mask images was not correlated with accuracy for real face images or with prior knowledge of hyper-realistic face masks. Image analysis revealed that mask and face stimuli were most strongly differentiated in the region below the eyes. Moreover, high-performing participants tracked the differential information in this area, but low-performing participants did not. Like other face tasks (e.g. identification), hyper-realistic mask detection gives rise to large individual differences in performance. Unlike many other face tasks, performance may be localized to a specific image cue.

## Significance 

The proliferation of Hollywood-style silicone masks has caught the security sector unawares. These whole-face masks allow wearers to transform their facial appearance in seconds and are readily accepted as real faces. The implications for security and crime prevention are potentially far-reaching, as undetected face masks undermine the connection between facial appearance and personal identity. Psychological research on face perception has discovered large individual differences in identification ability. The present studies similarly reveal large individual differences in the completely novel task of hyper-realistic mask detection and identify a specific region under the eyes that may drive accurate performance. Our findings raise the interesting prospect of selecting personnel for very narrow cognitive tasks. They also suggest that performance on this particular task may be responsive to training. Either route could improve our ability to distinguish hyper-realistic face masks from real faces.

## Background

**Fig. 1:**
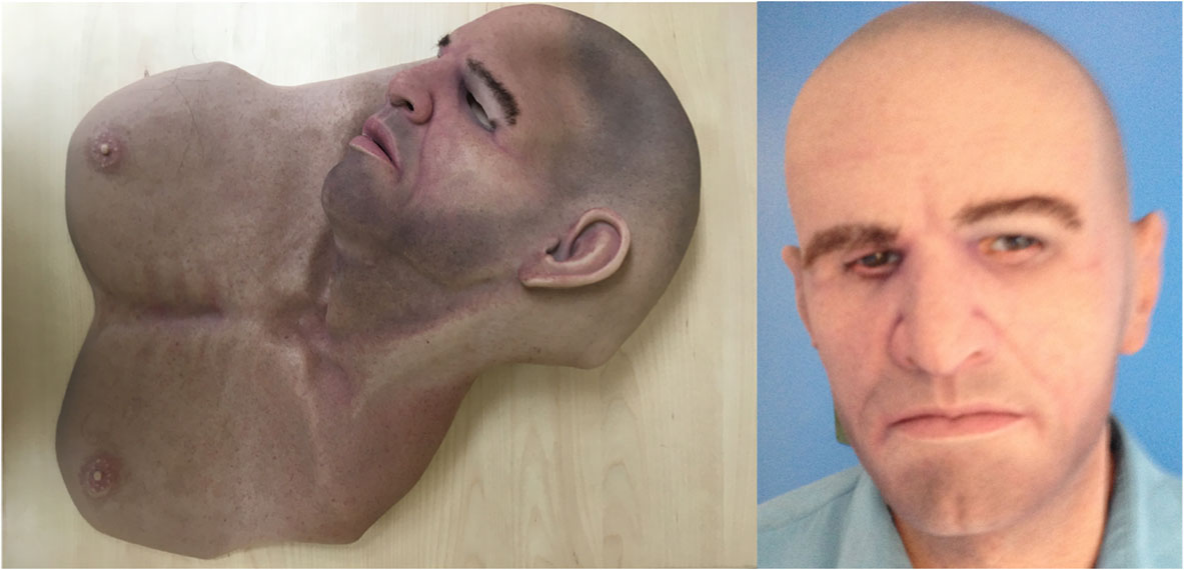
Hyper-realistic dominant male mask (*right*) worn by author RJ (*left*)

In a number of high-profile criminal cases, offenders have used hyper-realistic face masks (Fig. 1) to transform their facial appearance, leading police to pursue suspects who looked nothing like the actual offenders (e.g. different race or age; Bernstein, 2010). In a separate incident, an airline passenger wearing a hyper-realistic mask boarded an international flight without the deception being noticed (Zamost, 2010). These cases suggest that, in practical settings, hyper-realistic face masks can be difficult to distinguish from real faces. Experimental evidence bears out this conclusion. In a series of studies (Sanders et al., 2017), we examined incidental detection of unexpected but attended hyper-realistic masks in both photographic and live presentations. In all of these studies, viewers accepted hyper-realistic masks as real faces. These findings extend a tradition of research into realism of artificial stimuli. The Uncanny Valley phenomenon originally considered a range of human-like stimuli from puppets to robots (Mori, 1970; Mori, MacDorman, & Kageki, 2012). In recent years, the focus has shifted somewhat to computer-generated images (e.g. Nightingale, Wade, & Watson, 2017), but the very success of computer graphics has raised awareness that on-screen images may be digitally generated or enhanced. One of the interesting aspects of hyper-realistic masks is that they also fool the eye in the physical world (Sanders et al., 2017), where digital image manipulation has not yet encroached.

The finding that spontaneous mask detection is unreliable suggests that specific measures may be required if detection rates are to be improved. Here we pursue an individual differences approach to the problem. Over the last decade, individual differences have become an important topic in face perception research, not least because they suggest a route to improving performance in applied settings. For face identification, the range of ability is bracketed by two extremes. At the high end, super-recognizers who rarely make errors (Bobak, Bennetts, Parris, Jansari, & Bate, 2016; Robertson, Noyes, Dowsett, Jenkins, & Burton, 2016; Russell, Duchaine, & Nakayama, 2009), and at the low end, people with developmental prosopagnosia who rarely exceed chance performance (Behrmann & Avidan, 2005; Duchaine & Nakayama, 2005). Between these extremes, there is a spectrum of ability on standardized face identification tests (e.g. Burton, White, & McNeill, 2010; Duchaine & Nakayama, 2006).

These findings have led some researchers to suggest that personnel selection could play a useful role in optimizing occupational face recognition (White, Kemp, Jenkins, Matheson, & Burton, 2014). For example, Metropolitan Police super-recognizers have been found to score unusually high on a range of face identification tests (Robertson et al., 2016).

For mask detection, the cognitive situation is somewhat different. Here the challenge is not individuation at the subordinate level (Rosch, Mervis, Gray, Johnson, & Boyes-Braem, 1976), but rather categorization at the basic level, albeit for the unusual case where one basic category (masks) deliberately mimics the other (faces). As the current task involves face/non-face categorization, it arguably has more in common with face detection than with face identification (see Bindemann & Lewis, 2013, for a careful dissection of these issues).

The analogy with face detection may have some broad predictive value for the present case. Large individual differences in face detection ability have recently been reported (Robertson, Jenkins, & Burton, 2017) and they appear to dissociate from face identification ability. However, one important difference is that face detection hinges on the presence or absence of a face-like pattern (e.g. two eyes above a nose above a mouth). That criterion will not help the viewer in the current task, as hyper-realistic face masks and real faces both present face-like patterns. Thus, the intuition is that hyper-realistic mask detection will require finer discrimination than face detection tasks demand.

As yet, very little is known about individual differences in this finer perceptual task. For example, we do not know the expected range of ability. Nor do we know any factors that might differentiate high performers from low performers. The present studies address these issues by asking whether some people are better than others at categorizing masks and faces, and what they may be doing that allows them to perform well. The overarching aim is to establish whether an individual differences approach might be as useful in hyper-realistic mask detection as it has been in face identification.

We begin in Experiment 1 by comparing detection of low-realism and high-realism masks in the context of real faces. In Experiment 2, we eliminated low-realism masks to focus participants on the harder comparison (high-realism masks vs real faces). Finally, we undertook an image analysis to compare use of information for high- and low-accuracy subgroups.

## Experiment 1

Previous studies of hyper-realistic mask perception have assessed spontaneous detection of masks during an orthogonal task (social inference ratings; Sanders et al., 2017). Detection rates approached floor levels in that situation, precluding individual differences analysis. In this study, we sought to increase detection rates by: (1) explicitly instructing participants that the task was to distinguish masks from real faces; (2) presenting masks and faces equally often (50% prevalence); and (3) explaining this prevalence rate to participants. These measures were intended to license “mask” responses, even when participants were not certain. We expected that low-realism masks and real faces would be categorized accurately. Our main interest was in the range of performance for high-realism masks.

## Method

### Ethics statement

Ethics approval for all experiments was obtained from the departmental ethics committee at the University of York.

### Participants

Thirty members of the volunteer panel at the University of York (21 women, 9 men; mean age = 22 years, age range = 18–41 years) took part in exchange for a small payment or course credit.

### Stimuli and design

To collect images of high-realism masks, we entered the search terms “realistic masks,” “hyper-realistic masks,” and “realistic silicone masks” into Google Images. We selected images that: (1) exceeded 150 pixels in height; (2) showed the mask in roughly frontal aspect; (3) showed the eye region without occlusions; and (4) included real hair eyebrows. We used the same criteria to search the websites of mask manufacturers (e.g. RealFlesh Masks, SPFX, CFX) and topical forums on social media (e.g. Silicone Mask Sickos, Silicone mask addicts). Our aim here was to sample “ambient” photos of hyper-realistic masks that represent the range of the mask images in the visual world (Jenkins et al., 2011). For this reason, we avoided promotional studio photographs of the masks and instead used photos of the masks in situ. This search resulted in 37 hyper-realistic mask images that met the inclusion criteria.

For comparison, we collected 37 images of low-realism masks by entering search terms such as “Halloween,” “party,” “mask,” “masquerade,” “face-mask,” and “party mask” in Google Images and selecting the first images that met inclusion criteria 1–3 above.

**Fig. 2:**
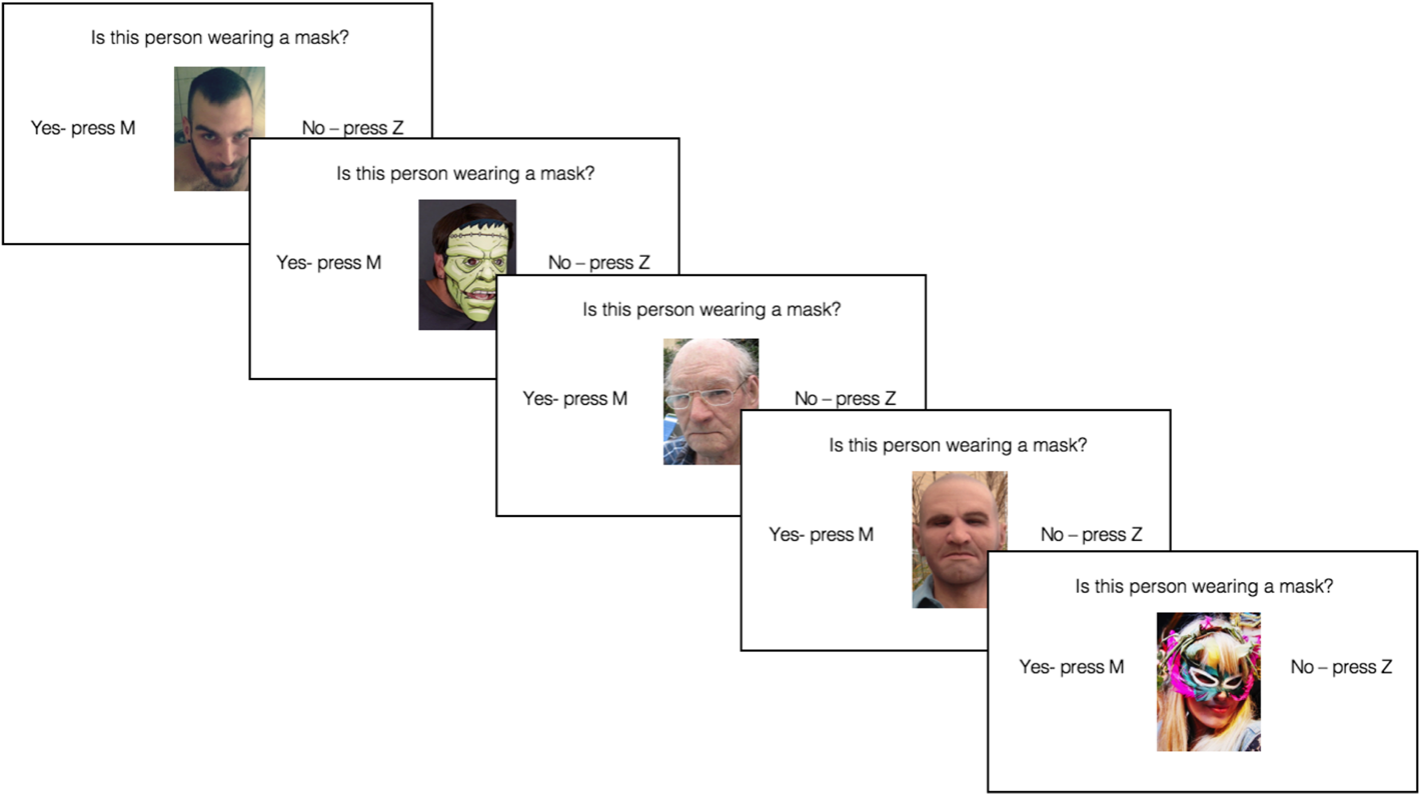
Example of trial sequence in Study 1. Correct responses: Z, M, M, M, M. See main text for details

We also collected 74 real-face images for use as fillers in the mask/face categorization task. To ensure that the demographic distribution among our real face images was similar to that portrayed by the high-realism masks, we entered the search terms “young male,” “old male,” “young female,” and “old female” into Google Images. We then accepted images that met criteria 1–3 until the distribution of faces across these categories was the same as for the high-realism mask images. All photos were cropped to show the head region only and resized to 540 × 385 pixels for presentation (see Fig. 2).

The final image set consisted of 148 photographs (37 high-realism masks, 37 low-realism masks, 74 real faces). Each participant viewed the 148 images intermixed in a different random order (within-subjects design).

### Procedure

Participants were instructed that half of the images showed real faces and half of the images showed masks. They were also informed that mask trials would contain both low-realism masks and high-realism masks. Each trial consisted of a centrally presented image (a mask or a face) together with the prompt “*Is this person wearing a mask?*” and response options “*Yes - Press M*” and “*No – Press Z*.” The display remained on screen until response, upon which the following trial began automatically. No time limit was imposed. Participants completed three practice trials, followed by 148 experimental trials in a unique random order. The entire experiment took approximately 10 min to complete.

## Results and discussion

### Group performance

**Fig. 3:**
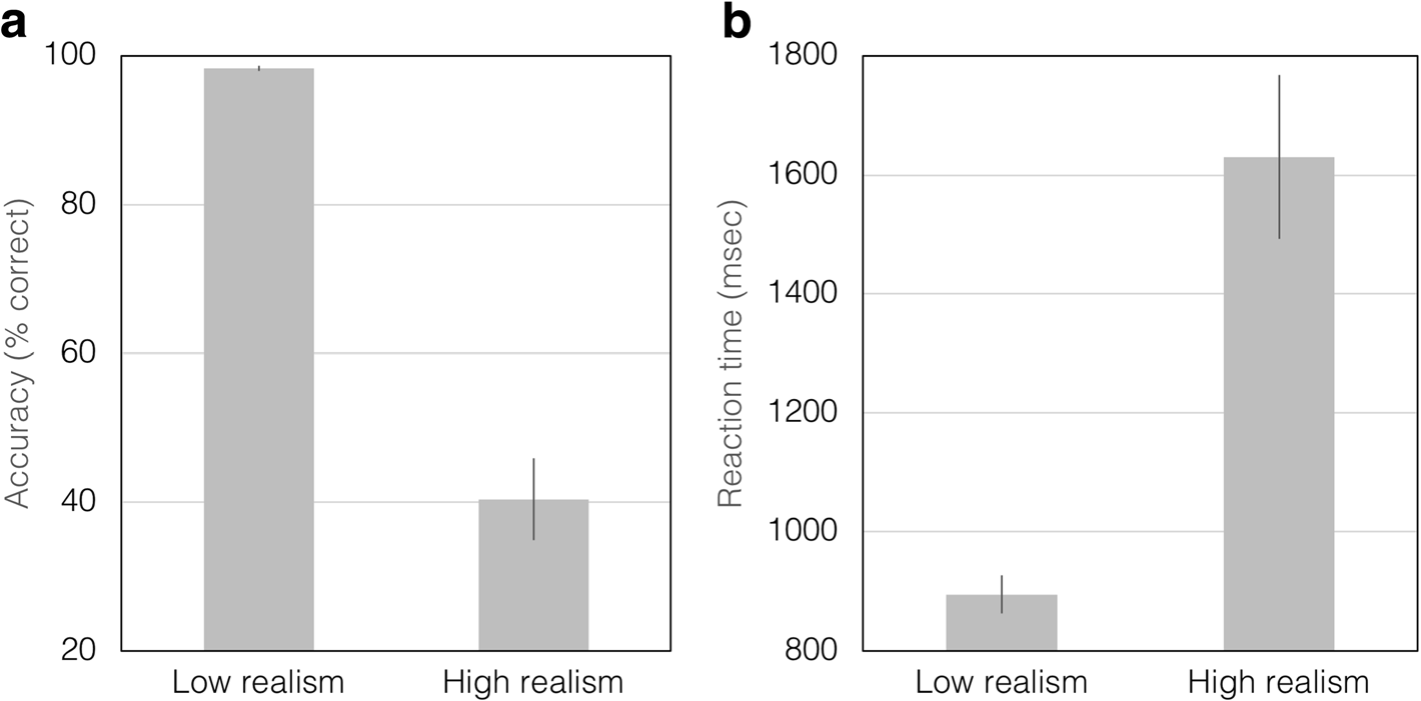
Mean accuracy rates (**a**) and correct reaction times (**b**) across participants as a function of mask condition in Study 1

Real face images were correctly classified on 96.3% of trials and were not analyzed further. Performance on mask trials is summarized in Fig. 3. As expected, low-realism masks were categorized reliably (M = 98.2%, SE = 0.4, CI = 97.6–99.0). High-realism masks were categorized much less reliably (M = 40.4%, SE = 5.6, CI = 29.2–51.5), meaning that the clear majority of these masks (59.6%) were misclassified as real faces. A within-subjects t-test confirmed that this difference in accuracy was statistically significant (t(29) = 10.29, *p* < 0.001).

Reaction time (RT) data followed a similar pattern. Correct responses to low-realism mask trials were relatively fast (M = 895 ms, SE = 35, CI = 831–959). Indeed, RTs to high-realism masks were twice as long 1629 ms (SE = 142, CI = 1352–1901). Again, the difference between mask conditions was statistically robust (t(29) = 5.86, *p* < 0.001).

### Individual differences

**Fig. 4:**
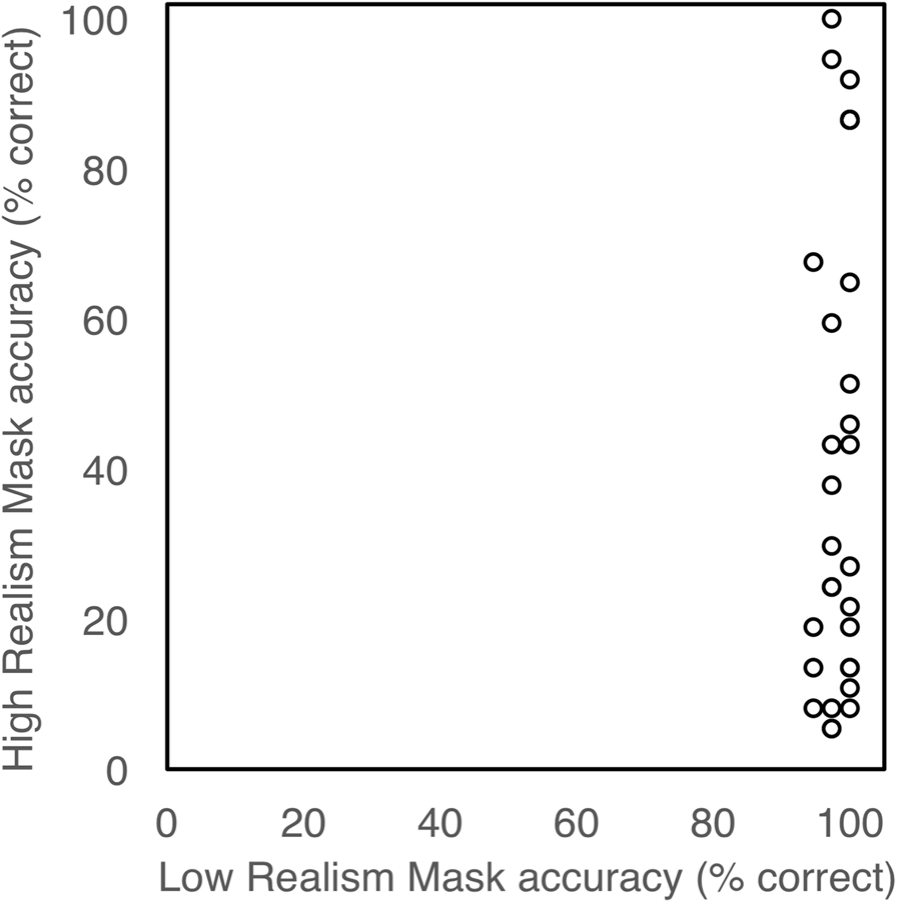
*Scatterplot* showing participants’ mean categorization accuracy rates in the high-realism and low-realism mask conditions in Study 1

As can be seen in Fig. 4, there was little variability in accuracy in the low-realism mask condition (range 95–100%), with performance compressed against ceiling for this easy task. In contrast, accuracy in the high-realism condition spanned the entire range (5–100%). Unsurprisingly, there was no correlation between high- and low-realism mask trial performance (*r* = 0.182, *p* = 0.335).

Overall, classification judgements were much harder for high-realism masks than for low-realism masks. More importantly for the current study, the data reveal striking individual differences in performance for the high-realism condition. A few observers detected hardly any hyper-realistic face masks in this experiment, but a few detected nearly all of them.

One possible interpretation of this pattern is that low-realism masks make high-realism masks hard to detect, by encouraging viewers to draw the category boundary in the wrong place ([*real faces + high-realism masks*] vs [*low-realism masks*] as opposed to [*real faces*] vs [*high-realism + low-realism masks*]). Prior knowledge of hyper-realistic face masks could protect against this error, leading to high overall accuracy. To address this possibility, we next repeated the experiment without the low-realism mask condition. We also asked participants whether they had encountered hyper-realistic face masks before the experiment.

## Experiment 2

This experiment was the same as Experiment 1, except for the following changes. First, we replaced the low-realism mask stimuli with high-realism mask stimuli, in order to focus participants on the difficult judgments (real faces vs hyper-realistic face masks). As before, we informed participants that half of the trials would contain real faces and half of them would contain masks. We expected the new composition of trials to elicit errors in both directions (i.e. masks mistaken for faces and faces mistaken for masks). Our main interest was the distribution of performance in this situation. To test for effects of prior mask knowledge on performance, we also collected self-report ratings at the end of the experiment.

## Method

### Participants

Thirty members of the volunteer panel at the University of York (24 women, 6 men; mean age = 20 years, age range = 18–24 years) took part in exchange for a small payment or course credit.

### Stimuli and design

Additional stimuli were collected via Internet search, using the method described in Experiment 1. Once again, the proportions of young male, old male, young female, and old female items were matched across real face and high-realism mask images. The final image set consisted of 148 photographs (74 high-realism masks and 74 real faces). Each participant viewed the 148 images intermixed in a different random order (within-subjects design).

### Procedure

**Fig. 5:**
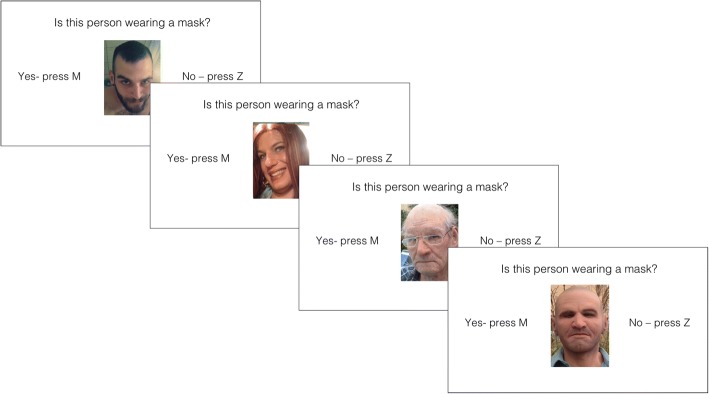
Example of trial sequence in Study 2. Correct responses: Z, Z, M, M

The procedure was the same as for Experiment 1, except that the low-realism trials were replaced with high-realism trials (see Fig. 5). To test whether individual differences in performance could be explained by prior knowledge of hyper-realistic face masks, we asked participants to rate their prior knowledge on a 7-point Likert scale at the end of the experiment.

## Results and discussion

### Group performance

**Fig. 6:**
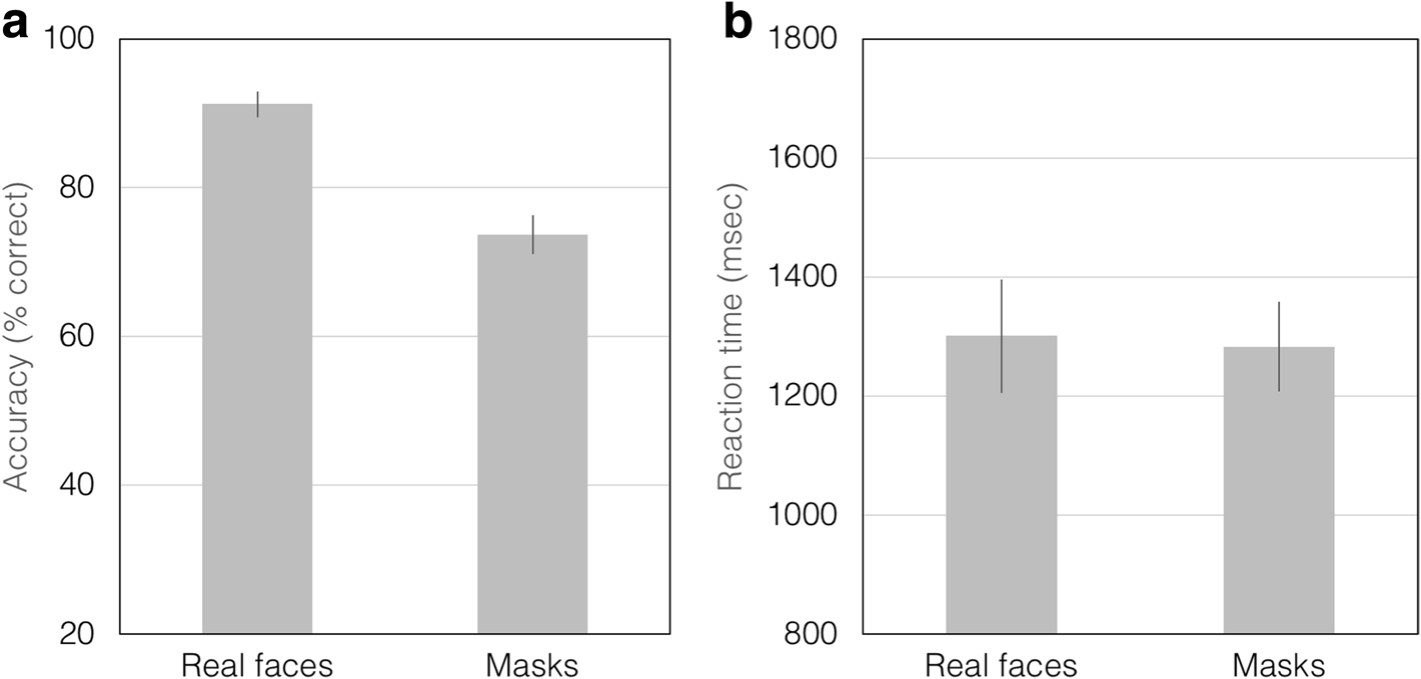
Mean accuracy rates (**a**) and correct reaction times (**b**) across participants as a function of experimental condition in Study 2

Overall categorization performance is summarized in Fig. 6. As can be seen from the figure, classification of real face images was accurate, but not at ceiling (M = 91.2%, SE = 2.0, CI = 87.3–95.1). Accuracy for high-realism masks was relatively low (M = 73.7%, SE = 2.7, CI = 68.3–79.0), indicating that hyper-realistic masks were frequently misclassified as real faces (26.3%). A within-subjects t-test confirmed that this difference in classification accuracy was statistically significant (t(29) = 6.78, *p* < 0.001).

To analyze discriminability and bias, we also carried out a signal detection analysis of correct performance (*d’ =* 1.56, SE = 0.43, CI = 1.40–1.72, *p*(correct) = 0.825). This indicates that participants were able to differentiate between masks and real faces. A criterion analysis (*C* = 0.61, SE = 0.09, CI = − 0.97 – – 0.43) indicates a modest bias towards responding “mask.” After correcting for this bias, the ability to discriminate masks from real faces remained (*d’ corrected =* 1.36, SE = 0.43, CI = 1.40–1.72, *p*(correct) = 0.854).

There was no significant difference in reaction times between real face (M = 1301 ms, SE = 93, CI = 1121–1480) and high-realism mask trials (M = 1283 ms, SE = 71, CI = 1145–1421; [t (29) = 0.34, *p* = 0.73]).

### Individual differences

**Fig. 7:**
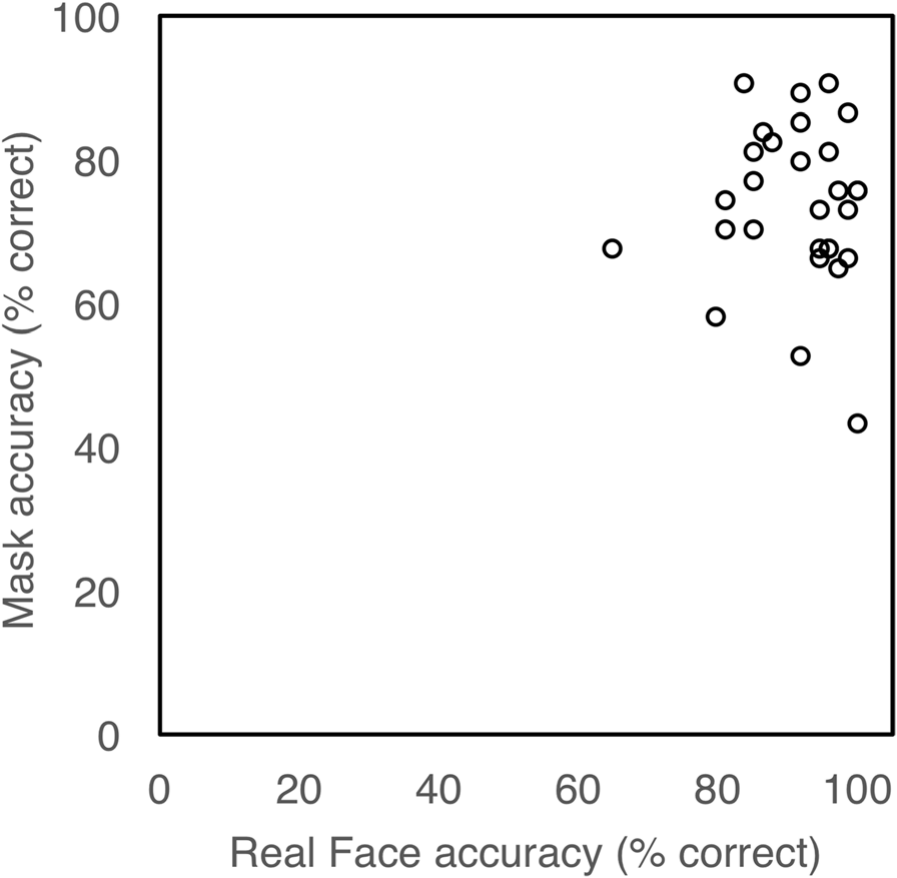
*Scatterplot* showing participants’ mean categorization accuracy rates in the real face and high-realism mask conditions in Study 2

As can be seen in Fig. 7, almost everyone performed above chance in both conditions. Classification accuracy was in the range of 65–100% in the real face condition and 43–91% in the high-realism mask condition. Interestingly, there was no correlation in performance between the two conditions (*r* = − 0.04, *p* = 0.83).

We also measured the individual differences in discriminability between masks and real faces in terms of sensitivity (d’: range = 0.59–2.34) and criterion scores (C: range = − 1.86 – 0.32).

### Prior mask knowledge

**Fig. 8:**
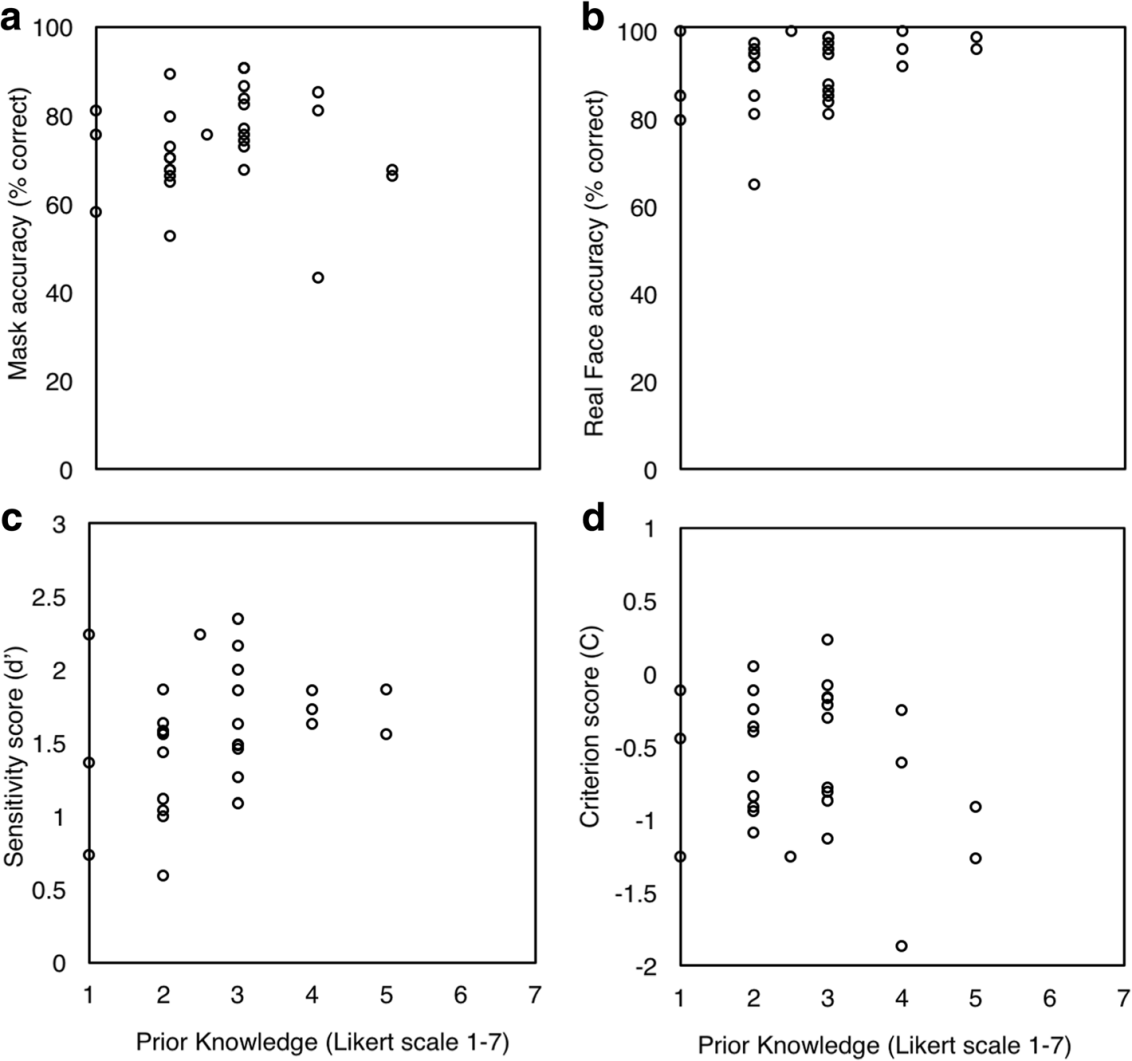
*Scatterplots* showing participants’ mean categorization accuracy rates for high-realism masks (**a**) and real faces (**b**), discriminability (**c**) and bias (**d**) as a function of prior mask knowledge in Study 2

Self-report ratings of prior mask knowledge were generally low (M = 2.67, SD = 1.03), suggesting little or no exposure to hyper-realistic face masks before the experiment. More importantly, there was no significant correlation between prior mask knowledge and performance in either the high-realism mask condition (*r* = 0.025, *p* = 0.898) or the real face condition (*r* = 0.319, *p* = 0.092), discriminability (*r* = 0.295, *p* = 0.120), or bias (*r* = − 0.218, *p* = 0.256) (see Fig. 8).

Overall error rates were high (20%) despite the simplicity of the task and despite the fact that participants were informed about the prevalence of mask and real face trials. We note that error rates were somewhat higher in the mask condition (30%) than in the real face condition (10%), meaning that overall, masks were mistaken for faces more often than faces were mistaken for masks. Interestingly, some participants were highly accurate in correctly categorizing the masks. However, accuracy in the mask condition was not explained by accuracy in the real face condition, nor by prior exposure to hyper-realistic face masks. In the final study, we ask whether high-performing individuals are using specific visual cues to support their accurate judgements.

## Image analysis

The purpose of the image analysis was to compare the use of visual information by high classification accuracy and low classification accuracy participants in Experiment 2. Our specific interests were: (1) the availability of visual cues—that is, whether mask and face images differed reliably; (2) the nature of any reliable visual cues—specifically, their spatial location; and (3) whether high-performing and low-performing participants made different use of these cues. We addressed these issues by using categorization data from Experiment 2.

The logic of this image analysis is as follows. The appearance of the mask stimuli and the face stimuli can be summarized by generating an average image for each stimulus category (an average mask and an average face). Systematic differences between these two categories can then be visualized by subtracting the average face from the average mask to create a difference image. This difference image indicates which regions of the stimulus are most informative for mask/face classification. Our hypothesis is that high-performing participants tracked this information more closely than low-performing participants. To test this hypothesis, we used categorization responses from Experiment 2 to generate difference images for the high-performing and low-performing subgroups. This allowed us to compare the *perceptual* difference images (based on participants’ categorization of the stimuli) against the *physical* difference image (based on the actual stimulus categories). By undertaking this comparison for different slices of the image, we were able to quantify participants’ tracking of category-level regularities across different face regions.

## Method

### Participant subgroups

To establish a strong manipulation of the independent variable (categorization accuracy for masks), we divided participants into performance quintiles (*N* = 6 per subgroup) and contrasted the highest and lowest quintiles. A 2 × 2 mixed ANOVA with the within-subject factor image type (mask, real face) and the between-subjects factor of subgroup (high, low) confirmed that these subgroups were statistically distinct with respect to their classification scores. Consistent with the whole-group analysis, we found a significant main effect of image type, with higher accuracy for real face trials (M = 90.0%, SE = 1.4, CI = 83.6–95.7) than for mask trials (M = 72.5%, SE = 1.5, CI 65.9–79.1), (F(1,10) = 13.76, *p* = 0.004, η2 = 0.58). More importantly, there was also a significant main effect of subgroup, with the high-accuracy group (M = 90.2%, SE = 0.8, CI = 86.7–93.8) reliably outperforming the low-accuracy group (M = 72.1%, SE = 2.1, CI = 62.9–81.2), (F(1,10) = 85.44, *p* < 0.001, η2 = 0.89). There was no significant interaction between these factors (F(1,10) = 1.78, *p* = 0.212).

### Face averages

**Fig. 9:**
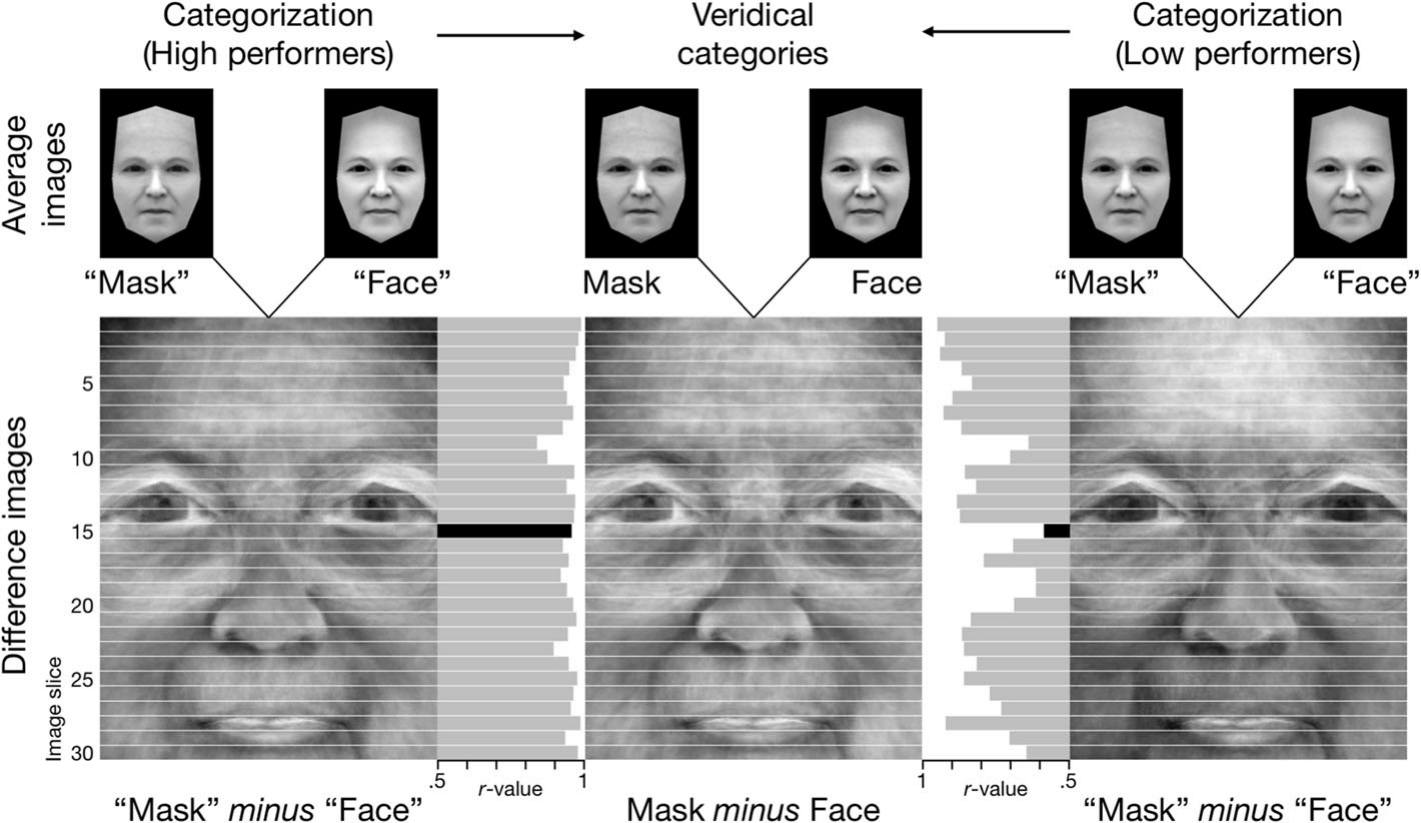
Summary image analysis in Study 3. Average images show mean pixel intensities across images in each category, separately for high performers (*left*), low performers (*right*), and veridical categories (*center*). Difference images are subtractions of pixel intensity (mask minus face; rescaled for visualization). Lighter *colors* indicate larger differences. Note the light region around the eye in the veridical difference image. The *y-axis* shows 30 horizontal image slices. Correlations between difference images (*gray bars*) are shown for each image slice. The largest discrepancy between high and low performers is shown at Slice 15 (*black bars*). High performers closely tracked categorial differences in this region. Low performers did not

We next constructed six average images (Burton, Jenkins, Hancock, & White, 2005) from the following six image sets: (1) actual masks (*N* = 74); (2) actual faces (*N* = 74); (3) perceived masks for high performers; (4) perceived faces for high performers; (5) perceived masks for low performers; and (6) perceived faces for low performers (weighted averages of images as classified; *N* > 50 for all). Seven images (five masks, two real faces) were excluded from this analysis because the camera angle did not allow accurate landmarking of the photographs (see Kramer, Jenkins, & Burton, 2017 for implementation details). The six weighted texture averages for the remaining images are shown in Fig. 9.

### Difference images

To ask what distinguishes masks from real faces, we next computed a difference image (average mask minus average face) separately for the veridical categories, the high-performance group, and the low-performance group. These three difference images are shown in Fig. 9 (lighter regions indicate greater difference). The veridical difference image (Fig. 9, center) indicates that the surrounding of the eye is especially informative, presumably because the eye holes in the mask can produce local anomalies in appearance (e.g. surface discontinuities if the mask is not flush with the wearer’s face; complexion discontinuities if the skin around the wearer’s eyes is exposed). The question is whether observers pick up on these subtle cues. Visual comparison confirms that the difference image for the high-performer group (Fig. 9, left) closely resembles the veridical difference image (Fig. 9, center). The difference image for the low-performer group (Fig. 9, right) resembles the veridical difference image less closely. This global pattern is perhaps to be expected, given the formation of the subgroups: if high performers did not track the veridical categories, they would not be high performers. However, local variations in this pattern may reveal specific cues that high performers exploit, and that low performers overlook. We investigated this possibility by comparing correlations between different image slices.

### Image correlations

To avoid spurious inflation of correlation values by black background pixels, we first cropped the background from each difference image to create rectangular face image (300 × 228 pixels) that retained all of the internal features. To allow direct comparison across equally sized regions, we then divided each rectangular image into 30 horizontal slices (10 × 228 pixels; see Fig. 9). Successive rows of pixels can be concatenated to form a single vector of pixels for each slice (1 × 2280 pixels), in which the grayscale intensity of each pixel is specified by an integer value between 0 (black) and 255 (white). These intensity values formed the input to the correlation analysis.

Figure 9 shows the results of these image correlations (r values), separately for each slice. As can be seen from the figure, correlations between the veridical difference image and the high-performer image are consistently high across image slices (range = 0.87–0.99). The correlations between the veridical difference image and the low-performer image are lower overall and much more variable (range = 0.59–0.95). Most strikingly, there is a distinct notch in correlation values between the low-performer and veridical difference images, directly under the eyes (image slice 15; *r* = 0.59). In fact, this was the lowest correlation in entire analysis. Importantly, that notch does not appear in the correlations between the high performer and veridical difference images (image slice 15; *r* = 0.95).

To summarize, our comparison of mask and face images suggests that the eye surround is the most informative region for separating these two categories. High performers appear to use information below the eye in a way that low performers do not. What information could be in this region? We suggest two possibilities. First, in a real face, the region below the eyes normally includes the lower eyelashes—an area of high local contrast. The masks in our stimulus set do not include eyelashes. If the mask covers the wearer’s eyelashes, it will typically reduce local contrast. Reduced local contrast under the eye may be a cue to mask detection. Second, in a real face, skin complexion below the eyes normally changes gradually on a local scale. The masks in our stimulus set do not necessarily match the complexion of the wearer. If the mask exposes any skin below the wearer’s eyes, it may cause an apparent discontinuity in skin coloration. Discontinuity in complexion under the eye may be a cue to mask detection. Each of these possibilities suggests that the precise fit of the mask around the wearer’s eyes is critical. Shade from the brow will tend to conceal cues in the upper eye region, at least under normal illumination conditions (light source above). However, the same illumination conditions will tend to highlight cues in the lower eye region, making them more salient.

## General discussion

Across three studies, we investigated individual differences in hyper-realistic mask detection—specifically, the ability to categorize images as masks or real faces. In Experiment 1, we found large individual differences in a mask/face categorization task for high-realism masks, low-realism masks, and real faces. Although low-realism masks (and real faces) were categorized accurately overall (> 98% correct), high-realism masks were not (40% correct). More importantly, from an individual differences perspective, accuracy in the high-realism condition ranged from floor (5%) to ceiling (100%), despite the consistently high accuracy for other stimulus types.

In Experiment 2, we discarded the low-realism mask condition to focus exclusively on the difficult categorization—hyper-realistic masks vs real faces. Perhaps surprisingly, removing the easy condition improved performance in the difficult condition considerably (74% correct). This seemingly paradoxical result underscores the importance of the context in which a categorization decision is taken. The absence of an obvious category distinction (cf. Experiment 1), combined with information about the distribution of stimuli, presumably led participants in Experiment 2 to approach the task differently. Nevertheless, we still observed a wide range of performance, even in this very different cognitive situation. Accuracy ranged from near chance (43%) to near ceiling (91%). Interestingly, accuracy in the real face condition was also varied (65–100%). However, performance in these two conditions was uncorrelated and was not explained by previous exposure to hyper-realistic face masks.

Both of these experiments revealed large individual differences in hyper-realistic mask detection, in the sense that some people were much more accurate than others at categorizing masks and real faces. These findings suggest that stable differences in ability may be worth pursuing. It is too early to say whether some individuals exhibit a special talent for this task. Conclusive evidence would require estimates of test–retest reliability and consistently high performance across a range of tasks (Russell et al., 2009; Robertson et al., 2016). Until then, we suggest another possible route to improved detection rates—one that does not depend on screening for high-aptitude individuals. In our image analysis, we asked what high performers are doing that low performers are not. This analysis revealed a candidate visual cue that these subgroups used differently—the area under the eyes. Hyper-realistic mask images and real face images diverged more strongly in this area than in other areas. Moreover, high performers and low performers diverged strongly in the extent to which the area under the eyes predicted their responses. This intriguing finding raises the question of whether mask detection could be improved by drawing attention to this region. If so, it could pave the way for a simple training intervention. This is a tantalizing prospect, especially as benefits of training in face identification tasks have proven difficult to pin down (Towler, White, & Kemp, 2014, 2017; White et al., 2014). Eye-tracking data in combination with accuracy rates, before and after training, should elucidate the potential of this approach.

Finally, it is worth returning to the somewhat artificial nature of this task. The experiment was specifically contrived to encourage detection of hyper-realistic masks. For example, we focused on masks in the task instructions and spelled out the distribution of mask and face stimuli. In view of this strong framing, the detection rate for these masks seems rather low. Nevertheless, it almost certainly overestimates the rate of spontaneous detection when a mask framing is absent. Sanders et al. (2017) reported extremely low rates of spontaneous detection, both for photographic presentations in the lab and live viewing of mask wearers outdoors. On the other hand, none of these studies has measured detection during active social interaction with the mask wearer (e.g. conversation). We expect that, in a more interactive context, additional cues from speech and movement could increase detection rate, but that is a matter for future studies.

Across these studies, we show that distinguishing hyper-realistic masks from real faces is a difficult task. Some people are much better than others at picking out hyper-realistic masks, and these large individual differences are not readily explained by correct categorization of real faces or by prior exposure to hyper-realistic masks. We suggest that they may be explained by differential use of specific visual cues and identify the region under the eyes as a promising candidate.

## Abbreviation

RT: Reaction time
